# Assessment of a Stream Gauge Network Using Upstream and Downstream Runoff Characteristics and Entropy

**DOI:** 10.3390/e21070673

**Published:** 2019-07-10

**Authors:** Hongjun Joo, Hwandon Jun, Jiho Lee, Hung Soo Kim

**Affiliations:** 1 Department of Civil Engineering, Inha University, Incheon 22212, Korea; 2 Department of Civil Engineering, Seoul National University of Science and Technology, Seoul 01811, Korea

**Keywords:** stream gauge network, entropy theory, unit hydrograph, runoff characteristic

## Abstract

A method for constructing a stream gauge network that reflects upstream and downstream runoff characteristics is assessed. For the construction of an optimal stream gauge network, we develop representative unit hydrographs that reflect such characteristics based on actual rainfall–runoff analysis. Then, the unit hydrographs are converted to probability density functions for application to entropy theory. This allows a comparison between two cases: one that considers the upstream and downstream runoff characteristics of a core dam area in South Korea, and another that uses empirical formula, which is an approach that has been widely used for constructing the stream gauge network. The result suggests that the case of a stream gauge network that considers upstream and downstream runoff characteristics provides more information to deliver, although the number of selected stream gauge stations of this case is less than that of the case that uses the empirical formula. This is probably because the information delivered from the constructed stream gauge network well represents the runoff characteristics of the upstream and downstream stations. The study area, the Chungju Dam basin, requires 12 stream gauge stations out of the current total of 18 stations for an optimal network that reflects both upstream and downstream runoff characteristics.

## 1. Introduction

A stream gauge network is an important water installation for the exact understanding of the amount of a water resource, because it provides basic water data [1]. A number of countries install or operate stream gauge stations for various purposes, including as part of flood alarm and warning systems, the operation of multipurpose dams, the analysis of available water resources, and the operation of agricultural reservoirs. Especially, current climate change and variability is offsetting precipitation globally, posing a dire threat to water resource management (WRM) [2]. Therefore, the importance of stream gauge stations is increasing day by day. However, issues can arise that limit the effectiveness of such systems, such as the uneven distribution of stations in a network area, and the lack of data sharing between stations, which lead to the duplication of gauging [3]. Due to this, countries are making continual efforts to establish a plan for producing and providing diverse hydrological information on a real-time basis.

To assess stream gauge stations installed for different purposes, entropy, principal component regression analysis, and correlation analysis are used. Entropy is most used for the assessment of existing gauge networks [4,5,6,7,8]. The principle of entropy has been used for a range of research fields, including the hydrological assessment of gauge networks. For instance, Caselton and Husain [4] applied the concept of information delivery to designing a hydrological network, while Chapman [5] estimated the uncertainty of hydrological data by using entropy and applying the data to modeling for the assessment of the decrease in uncertainty. Yang and Burn [9] designed an optimal gauge network based on entropy methodology, while Al-Zahrani and Husain [7] applied it to providing the optimal number of stations for a dense network and an extension plan for a sparse network. In addition, Markus et al. [8] extended the principle of entropy and generalized least square (GLS) methodology to assessing stream gauge stations located in Illinois, in the United States (U.S.A).

Entropy theory was also applied to rain gauge networks. Kim et al. [10] evaluated network density and spatial distribution based on the theory to conduct spatial correlation analysis. Lee et al. [11] proposed a plan for network optimization using a multipurpose genetic algorithm that considered both the quantity and the importance of the information delivered from rain gauge networks.

In addition, entropy is a useful theory for diverse fields related to water resources. Singh [12] suggested a hydrological process for parameter estimation, based on the principle. Chou [13] used multiscale entropy (MSE) to provide a new methodology for analyzing the complexity of runoff coefficients on rainfall, runoff, and time. Zhu et al. [14] introduced entropy to explain two types of influence, namely, climate change and anthropogenic interventions, on the evolution process of the water resource system. Wrzesiński [15,16] and Faiz et al. [17] assessed the uncertainty of flow regime characteristics and precipitation variability using entropy theory.

Entropy has been widely used for constructing hydrological gauge networks, and when it comes to constructing an optimal gauge network, utmost importance is given to its relevance to the purpose of network installation. It is the best case that a hydrological station is established densely. However, budgeting is necessary to operate many hydrological stations. An optimal configuration of stream gauge stations is essential to improve the resource utilization and increase cost savings in a hydrologic monitoring network [18]. In terms of maintenance, it is efficient to operate the least number of stations. For that, the entropy theory is mainly applied to evaluate the observation network.

Stream gauge networks constructed with entropy will vary, depending on the characteristics of input data related to the network. In this study, a representative unit hydrograph was applied as input data, and its characteristics determined the constructed network. The representative unit hydrograph should well reflect the runoff characteristics of the areal points of water gauge stations. Moreover, it should be noted that stream gauge stations have close relations to each other, unlike rain gauge stations. The runoff of stream gauge stations is under the direct influence of the stations at the upper part of the stream, and each of the stations cannot be independently approached. For the construction of an optimal stream gauge network for a target area, the close correlations between upstream and downstream runoff characteristics as well as the hydrological similarities between stations should therefore be considered, rather than assessing the stations individually. The correlation analysis of hydrological data between stations is an important process in the design of the network [19], and has also been applied in the environmental field [20].

With this in mind, our study assessed stream gauge networks with a focus on how to construct them in a way that they reflect upstream and downstream runoff characteristics. To create an optimal network, the study developed representative unit hydrographs that reflect upstream and downstream runoff characteristics, which were based on actual rainfall analysis. Then, they were converted to probability density functions to be applied to entropy. This allows for a comparison between two cases: one that considers the upstream and downstream runoff characteristics of the Chungju Dam area in South Korea, and another that uses empirical formula, with the ultimate goal of acquiring stable stream data.

## 2. Entropy to Assess a Stream Gauge Network

Entropy is widely recognized as an indicator of randomness or uncertainty. For the field of information theory, it is defined as the information capacity that a signal has [21]. In other words, when a signal is sent to the process of information delivery, the signal’s uncertainty decreases with information increasing to a level enough to eliminate the uncertainty. Therefore, the level of decreased uncertainty can be used to indirectly estimate the information on a signal [22]. Shannon and Weaver [21] defined the marginal entropy on a discrete random variable X as:

(1) $$H\left(X\right)=-\overset{N}{\underset{n=1}{\sum }}P\left(x_{n}\right)\ln P\left(x_{n}\right),\;\;\;\;\;\;n=1,2,3,\ldots ,N$$

where $P\left(X_{n}\right)$ is the occurrence probability of $X_{n}$, and H(x) indicates the information quantity or uncertainty that X has. When $y_{m}\left(m=1,2,\ldots ,N\right)\;$is given in relation to the random variable $X_{n}$, the estimation of $X_{n}$ from $y_{m}\;$can decrease the uncertainty that $X_{n}$ has. This principle can produce the variable, Y, and the uncertainty that remains in the random variable, X, due to Y can be estimated as:

(2) $$H\left(X|Y\right)=-\overset{N}{\underset{n=1}{\sum }}\overset{N}{\underset{M=1}{\sum }}P\left(x_{n},y_{m}\right)\ln P\left(x_{n}|y_{m}\right)$$

where $P(x_{n}|y_{m})$ is the joint probability of $X=\left(x_{n}\right)$ and $Y=\left(y_{m}\right)$, while $P(x_{n}|y_{m})$ is the conditional entropy of X to the given Y. $P\left(x_{n},y_{m}\right)$ also indicates the quantity of information lost in the process of information delivery between X and Y [8]. The level of decrease in the uncertainty that remains in X by the given Y or the quantity of information delivery (information sharing and overlapping) between X and Y can be obtained as:

(3) T(X,Y)=H(X)−H(X|Y)

Currently, probability density functions that can be applied to the entropy method are limited to normal distribution, lognormal distribution, and gamma distribution. This is because theoretical entropy values are given and easily used only for these three types of distribution, but for the others, complex, multidimensional, and numerical integration is required to apply them to the method [9]. The concept of entropy can be applied to hydrological time series data. If it is assumed that the continuous random variable, X, follows probability density function, f(x), the range of X can be divided in the interval of Δx. Chapman [5] applied Δx/x, which is the interval proportional to the range of the variable, instead of the fixed interval, Δx, and defined the marginal entropy and the conditional entropy, respectively, as:

(4) $$H\left(X;\Delta x\right)=u+0.5\ln\left(2\pi e\sigma_{z}^{2}\right)-\ln\left(\Delta x\right)$$

(5) $$H\left(X|Y;\Delta x\right)=u+0.5\ln\left[\left(2\pi e\sigma_{z}^{2}\right)\left(1-\rho_{zw}^{2}\right)\right]-\ln\left(\Delta x\right)$$

(6) $$T\left(X,Y\right)=-0.5\ln\left(1-\rho_{zw}^{2}\right)$$

where, $u_{z}$ and $\sigma_{z}$ indicate the average and the standard deviation (SD) of z(=lnx), respectively. $\rho_{zw}$ is the correlation coefficient of z and w(=lny). The optimization of a gauge network for a basin means reducing the number of gauge stations to minimize the overlapping information between stations in addition to securing the most information on the basin from the remaining stations. In other words, it means acquiring the most information on the basin from the least number of stations. In this regard, the objective function for optimization can be expressed as [7]:

(7) $$MAX\;\left[T\left(X_{1},X_{2},\ldots X_{m};X_{k},X_{l},\ldots X_{p}\right)\right]$$

where m indicates the total number of existing gauge stations in the basin, while p is the number of stations that should remain. $T\left(X_{1},X_{2},\ldots X_{m};X_{k},X_{l},\ldots X_{p}\right)$ represents the amount of information about a basin that can be obtained from p number of gauge stations:

(8) $$MAX\;\overset{m}{\underset{i=1}{\sum }}T\left(X_{i};X_{k},X_{l},\ldots X_{p}\right)=MAX\left(H\left(X_{k}\right)+H\left(X_{p}\right)+\overset{m-p}{\underset{i=1}{\sum }}\overset{p}{\underset{j=k}{\sum }}T\left(X_{i},X_{j}\right),\;i\neq j\right)$$

$H\left(X_{k}\right)+H\left(X_{p}\right)$ adds up the marginal entropy of each selected gauge station, and $\overset{m-p}{\underset{i=1}{\sum }}\overset{p}{\underset{j=k}{\sum }}T\left(X_{i},X_{j}\right)\;$represents the information delivery between selected and non-selected gauge stations, or the information content about non-selected gauge stations that can be obtained from selected gauge stations. With an increasing number of stations, the information that can be acquired also increases. However, after a certain point in time, the information from selected stations decreases due to overlapping information between stations. Therefore, an optimal gauge network means a combination of stations that can generate the maximum quantity of information.

## 3. Study Area

### 3.1. Selected Study Area

**Figure 1:**
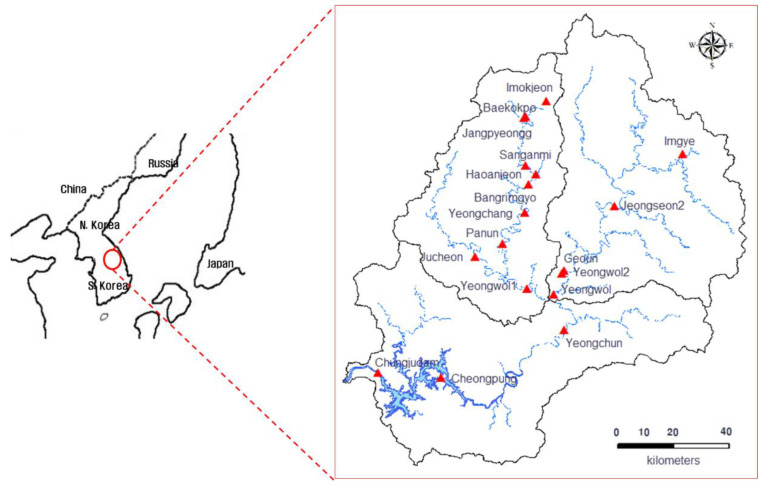
Locations of the stream gauge network and its stations in the study area.

The target area of this study consists of the Namhan River basin, Pyeongchang River basin, and Chungju Dam basin. The area is located in the central eastern part of the Korean Peninsula, ranging from a latitude of 37°41′ to a longitude of 128°02′–129°01′. This region includes some parts of the Chungcheong and Gangwon provinces, for which the importance of stream gauges has been highlighted in estimating the change in flow rate of the Han River: the river that best represents South Korea. For the target area, the Namhan River basin and the Pyeongchang River basin were considered as upstream, while the Chungju Dam basin was considered as downstream, with a total basin area of 6649.1 km^2^ and a stream length of 254.9 km. As of 2019, there are 29 stream gauge stations in operation, but only 18 of them were selected for analysis, as 11 of the stream gauges only observe water level. The main purpose of this study is to propose a plan for improving the existing calculation methodology for unit hydrographs and constructing a gauge network that reflects upstream and downstream runoff characteristics. Figure 1 shows the locations of the stream gauge stations that were covered by this study.

### 3.2. Density of Stream Gauge Stations in the Study Area

When evaluating the stream gauge stations, whether a sufficient number of stations have been established within the basin and whether they are appropriately established to satisfy the purpose of the stream gauge stations must first be considered. The density of stream gauge stations suggested by the WMO (World Meteorological Organization) is used to determine whether there is a sufficient amount of stream gauges installed in the basin. Table 1 summarizes the area’s topographical characteristics and density of each stream gauge station.

There are a total 18 stream gauge stations established in this study area, and the density is 47.9–841.1 km^2^/stream gauges. Table 2 shows the minimum densities of the stations depending on the stream gauge type suggested by the WMO [23].

This study area corresponds to the mountain area. So, the stream gauge stations satisfy the minimum installation standard suggested by the WMO [23]. However, despite meeting the minimum installation standard, it is difficult to say whether the established stream gauge stations are uniformly dispersed [24]. While the minimum density of the stream gauge stations suggested by the WMO makes it difficult to evaluate the spatial distribution characteristics, it is the minimum standard for collecting stream gauge data.

## 4. Development of Unit Hydrographs and their Conversion to the Probability Density Function

### 4.1. Rainfall–Runoff Analysis for the Development of Unit Hydrographs

Unit hydrographs are obtained through the analysis of observation data or using empirical formula. The Clark unit hydrograph is the most suitable method to estimate the amount of runoff in Korea. This method requires two parameters: concentration time and storage coefficient [25]. When no observation data are available, empirical formulas are used to calculate the parameters. Most of the empirical formulas are calculated with topographical characteristics. However, when observation data are available, parameters are determined by rainfall–runoff analysis. This study used observed data to develop a unit hydrograph, and then evaluated a gauge network by comparing it with the result obtained with the methodology for unit hydrograph development suggested in previous research [26]. As mentioned above, the purpose of using observed data for the development of a unit hydrograph is to reflect the runoff characteristics that are different between upstream and downstream data as well as from station to station.

At the outset of the development of a unit hydrograph, sub-basins should be set for each stream gauge. Then, it is necessary to obtain topographical factors for each of the sub-basins before the estimation of parameters of runoff characteristics by sub-basin. This study related two models, HEC-HMS (hydrologic engineering center-hydrologic modelling system) and HEC-GeoHMS (hydrologic engineering center - geospatial hydrologic modelling system), to construct sub-basins.

**Figure 2:**
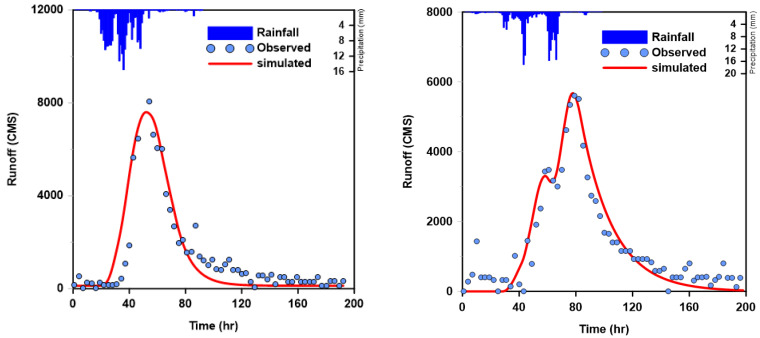
Rainfall–runoff analysis result for parameter verification and calibration.

For the analysis of rainfall–runoff, the subdivision of the basin that has a stream gauge station into outlets should be preceded in order to determine the runoff characteristics through a comparison between the runoff analysis model and the observed data. To construct the HEC-HMS model, a rainfall loss process, basin routing method, and channel routing method are required. The NRCS (Natural Resources Conservation Service) method is applied to the rainfall loss process, the Clark unit hydrograph method for basin routing, and the Muskingum method for channel routing. For the initial values, to calculate the parameters of the Clark unit hydrograph, the Kraven (II) formula and the Sabol formula were used. The Thiessen method was used to obtain the areal average rainfall. In addition, the Antecedent Moisture Condition (AMC-III) was applied to maximize the flood peak. The observed rainfall events in the period from June to September (rainy season), where the flood peak is usually high, were selected, and the parameters were verified and calibrated with the observed flow values, as explained above. Table 3 and Figure 2 show the results of rainfall–runoff analysis, for which the study completed verification and calibration by using the rainfall events applied to the optimization of the parameters and inflow data for the target area.

### 4.2. Development of Unit Hydrographs by Gauge Station

Real runoff curves are dependent on the rainfall pattern. It is difficult to convert the real runoff curve to a probability density function, because the real runoff curve has an irregular pattern. The unit hydrograph of a watershed is defined as a direct runoff hydrograph resulting from one unit of excess rainfall generated uniformly over the drainage area at a constant rate for an effective duration. For the construction of an optimal gauge network using entropy, unit hydrographs to be converted to probability density functions are required. For Case 1, unit hydrographs were derived with the observed rainfall–runoff events. Case 2 based on the empirical formulas is applied when there is no observation data.

When applying the empirical formula, it is assumed to be a merged basin based on the outlet. The runoff characteristics at the outlet are the integrated result of the characteristics of the upstream (steep basin) and the downstream (mild slope). It becomes more difficult to consider the influence of the tributaries when applying empirical equations. These cases can be seen in two types of basins. In the first case, two sub-basins of the same size join together at confluence point (two sub-basins located in parallel). At the confluence point, the watershed area is doubled, but the time of concentration (Tc) is determined by selecting a larger longest flow path of the two sub-basins. When estimating the unit hydrograph at the confluence point, the influence of the tributary stream (the small Tc) is not taken into consideration, because it is assumed to the merged basin at the confluence point. The second case is that the upstream basin (B) is located in the downstream basin (A). The unit hydrograph of the downstream should be estimated with the Tc, including the upstream basin (B). In Case 2, in which both streams are integrated into one basin, only the average Tc is calculated, even if the same stream are made of a steep slope stream and a mild slope stream. That is, it is assumed that Tc is assumed to be the same flow rate by the stream average slope, although the runoff characteristics of the upstream basin (B) are included. Therefore, it is difficult to consider clearly the basin characteristics of the upstream basin (B), because it is assumed to the merged basin at the confluence point. Therefore, Case 2 can be used to derive the runoff characteristics for each sub-basin, but it does not consider the runoff relationships between sub-basins. On the other hand, when actual rainfall–runoff data (Case 1) is used, the river connectivity between upstream and downstream can be considered, since the discharge of the tributary is assumed as the inflow of the main stream.

**Figure 3:**
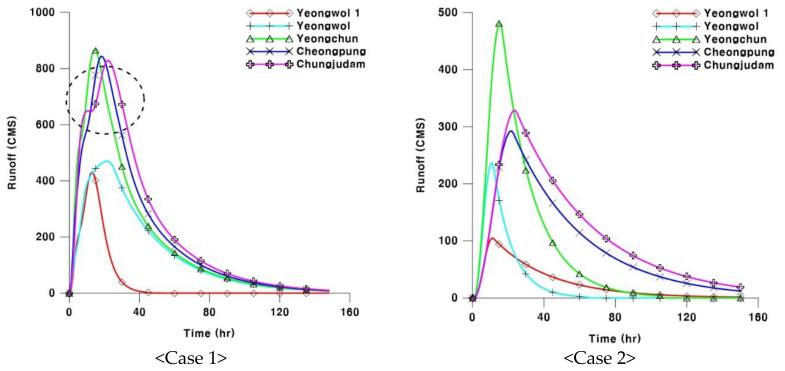
Comparison of the developed unit hydrographs between the two methodologies.

Case 1 takes into account the stream connectivity and reflects the real runoff phenomenon. The above procedure is important to decide the parameter of lognormal distribution. For Case 2, unit hydrographs were calculated with empirical formula, as applied in a previous study [26]. When empirical formulas are used, actual events between the sub-basins are not considered; instead, only the individual characteristics of the watershed are considered. Previous study [26] applied the Kirpich empirical formula for the time of concentration, and the Sabol empirical formula for the storage constant. Figure 3 shows the calculation results of unit hydrographs using the two formulas for different points of the target area: Yeongwol (downstream of the Namhan River); Yeongwol 1 (downstream of the Pyeongchang River basin); Yeongchun and Cheongpyeong (the junction of the two rivers); and Chungju Dam (the lowest downstream section of the study area).

For the two cases, unit hydrographs were calculated. As a result, the unit hydrographs developed on the basis of rainfall–runoff events (Case 1) did not show a curve as gentle as that in Case 2 (see Figure 3). This was the result of the runoff characteristics varying between the upper stream and the downstream. This phenomenon occurs as the runoff of a downstream section is directly influenced by the characteristics of the upper stream’s runoff characteristics. In addition, Chungju Dam in the lowest downstream section had a lower flood peak compared to its upper sections, Yeongchun and Cheongpyeong, due to topographical influence. In other words, at the Yeongchun and Cheongpyeong points, which have a high share of steep slopes, the flood peak sharply increased, but at the same time, discharged rapidly. Meanwhile, the Chungju Dam basin, which has a gentle slope and a slow flow rate, showed a significant effect of the channel storage, which led to the low flood peak and the slow discharge. Therefore, Yeongchun had the highest peak flow, followed by Cheongpyeong and the Chungju Dam.

Case 2, in which the unit hydrograph is obtained with the empirical formula, did not reflect upstream and downstream runoff characteristics. For this reason, the peak flow of the Chungju Dam was found at the midway point between Yeongchun and Cheongpyeong. In addition, the runoff of Case 2 showed a significant difference to that of the flow outlets: the Chungju Dam and Yeongchun. For the Chungju Dam and Yeongchun, the concentration time was calculated with the empirical formula as 15 hours and 23 hours, respectively, while the storage constant was 18 hours and 44 hours, respectively. The calculation results are considered as having high relevance because the channel distance between the two points is about 87 km, and the travel time for the distance is estimated as 7 hours, with an assumed flow rate of 3.5 m/s for the flood season.

The concentration time can be obtained simply with the characteristic factors of the basin for parameter calculation. The storage constant, on the other hand, requires a relatively complex process to estimate the parameter. For Sabol, an empirical formula commonly used in Korea, river length, and area calculations are needed to reflect the basin shape. Therefore, its application is limited to the basin of a less meandering stream with a long bar shape; otherwise, the accuracy of storage constant calculation is low [27]. When calculating the storage constant, more attention should thus be given to the application of empirical formula compared to when calculating the concentration time. In particular, for the case where two basins are joined, the higher river length value of the two is selected. This leads to the result of a significantly increased basin area, but no change in its length. For basins with runoff characteristics that are common in Korea, it is known that storage constants are about 0.8–1.2 times higher compared to the concentration times. For this reason, the Chungju Dam showed a significantly smaller peak flow than Yeongchun due to the high value of the storage constant at its lower downstream section. In addition, to generate data for rainfall–runoff analysis and flood prediction, which is the main purpose of stream gauge stations, it is important to design a network that reflects upstream and downstream runoff characteristics, as in Case 1.

### 4.3. Conversion of the Developed Unit Hydrograph to the Probability Density Function

**Figure 4:**
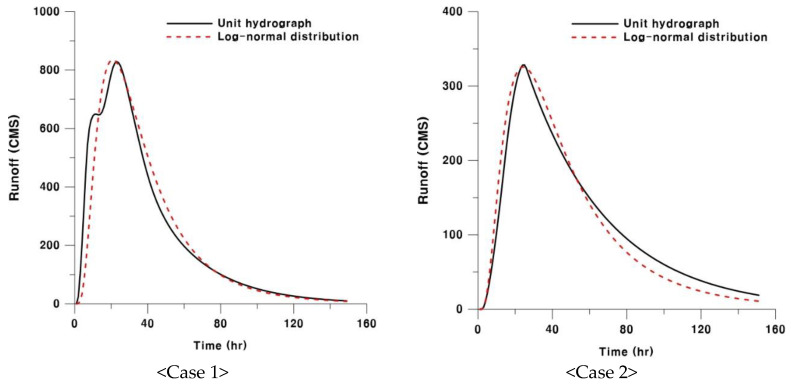
Matching up the unit hydrograph and the lognormal distribution (Chungju Dam station).

The main objective of this study is to assess two cases for developing unit hydrographs and conduct a comparative analysis with a special focus on a gauge network constructed with each of the two methodologies. One case is based on an existing empirical formula, and the other case is based on upstream and downstream runoff characteristics. A gauge network construction using entropy requires a probability density function that well represents unit hydrographs developed for each of the points in the target area. The probability distribution types used for hydrological data analysis are roughly divided into two groups: discrete distribution and continuous distribution. For the former type, binomial distribution and Poisson distribution are commonly used to determine time intervals to trigger a certain intensity of rainfall and flood or a probability of event occurrence. Nonetheless, as most hydrological events occur on a continuous basis, the latter type, continuous distribution, is mainly applied in such probabilistic analysis. Among such continuous distribution types, normal distribution, lognormal distribution, gamma, log Pearson, and generalized extreme value (GEV) distribution are the most widely used for hydrological analysis. Entropy formulas that are in accordance with the probability density function include normal distribution, lognormal distribution, and gamma distribution. Zhang and Singh [28] and Guo et al. [29] converted the probability density function to apply entropy theory. However, it is difficult to apply other distributions to the unit hydrographs except for the lognormal distribution. This study used lognormal distribution, because it was considered the most suitable for the unit hydrographs developed for each of the sub-basins. Equation (9) represents the equation of the lognormal distribution:

(9) $$f\left(x\right)=\frac{1}{xb\sqrt{2\pi }}\frac{1}{x}\exp\left[-\frac{1}{2}{\left(\frac{lnx-\mu_{y}}{\sigma_{y}}\right)}^{2}\right],\;0\leq x<\infty$$

where lnx=y, μ = average, and σ = SD (Standard Deviation). A previous study [23] estimated the concentration times and storage constants, which were based on empirical formulas, before calculating the parameters of the lognormal distribution by using a moment method. It is common to apply such a method to estimate the parameters, but this study used an optimization method using a genetic algorithm based on Visual Basic to provide more accurate parameters. Table 4 shows the result of the comparison of the parameters between the two probability density functions: one is suggested when using an empirical formula, and another was estimated through rainfall–runoff analysis. Then, Figure 4 illustrates the results of matching up the unit hydrograph and the lognormal distribution for sub-watersheds.

## 5. Application of the Entropy Theory and Construction of an Optimal Stream Gauge Network

In this study, unit hydrographs were applied for two separate cases: one using an empirical formula, and the second by reflecting the runoff characteristics of the upstream and downstream gauge stations. In this section, these unit hydrographs become converted to probability density functions, which are applied to entropy for the construction of optimal gauge networks for the comparison of the two case approaches. First, for the application of entropy, an information delivery matrix should be generated. Table 5 and Table 6 show the summarized results of the information delivery matrix for each of the two cases. The information delivery matrices presented in Table 5 and Table 6 indicate the quantity of information delivered between stations. The lines through the matrices show the marginal entropy of each station.

An optimal stream gauge network constructed using entropy means that its stations should provide the maximum information of the runoff from its stations in the basin. Thus, an optimal network minimizes overlapping information between stations, and produces as much basin information as possible from selected stations. The information is delivered between points, and the total quantity of information means the total delivered information on the basin from a selected point. If only one station is selected, station 10 (5.248) and 13 (5.638) can be selected for cases 1 and 2, respectively. When the gauge stations are combined, the total entropy information for each station can be estimated from Table 5 and Table 6 using Equation (8) as follows.

□ Case 1
  $$T\left(X_{1};X_{2},X_{3},\ldots X_{18}\right)=H\left(X_{1}\right)+T\left(X_{1},X_{2}\right)+T\left(X_{1},X_{2}\right)+\cdots \;T\left(X_{1},X_{18}\right)]=\;7.40\;T\left(X_{2};X_{1},X_{3},\ldots X_{18}\right)=H\left(X_{2}\right)+T\left(X_{2},X_{1}\right)+T\left(X_{2},X_{3}\right)+\cdots \;T\left(X_{2},X_{18}\right)]=\;14.00\;\vdots \;T\left(X_{10};X_{1},X_{2},\ldots X_{18}\right)=H\left(X_{10}\right)+T\left(X_{10},X_{1}\right)+T\left(X_{10},X_{2}\right)+\cdots \;T\left(X_{10},X_{18}\right)]=\;16.12\;\vdots \;T\left(X_{18};X_{1},X_{2},\ldots X_{18}\right)=H\left(X_{18}\right)+T\left(X_{18},X_{1}\right)+T\left(X_{18},X_{2}\right)+\cdots \;T\left(X_{18},X_{17}\right)]=\;9.02$$
□ Case 2
  $$T\left(X_{1};X_{2},X_{3},\ldots X_{18}\right)=H\left(X_{1}\right)+T\left(X_{1},X_{2}\right)+T\left(X_{1},X_{2}\right)+\cdots \;T\left(X_{1},X_{18}\right)]=\;8.80\;T\left(X_{2};X_{1},X_{3},\ldots X_{18}\right)=H\left(X_{2}\right)+T\left(X_{2},X_{1}\right)+T\left(X_{2},X_{3}\right)+\cdots \;T\left(X_{2},X_{18}\right)]=\;10.00\;\vdots \;T\left(X_{13};X_{1},X_{2},\ldots X_{18}\right)=H\left(X_{13}\right)+T\left(X_{13},X_{1}\right)+T\left(X_{13},X_{2}\right)+\cdots \;T\left(X_{13},X_{18}\right)]=\;14.60\;\vdots \;T\left(X_{18};X_{1},X_{2},\ldots X_{18}\right)=H\left(X_{18}\right)+T\left(X_{18},X_{1}\right)+T\left(X_{18},X_{2}\right)+\cdots \;T\left(X_{18},X_{17}\right)]=\;8.72$$

**Figure 5:**
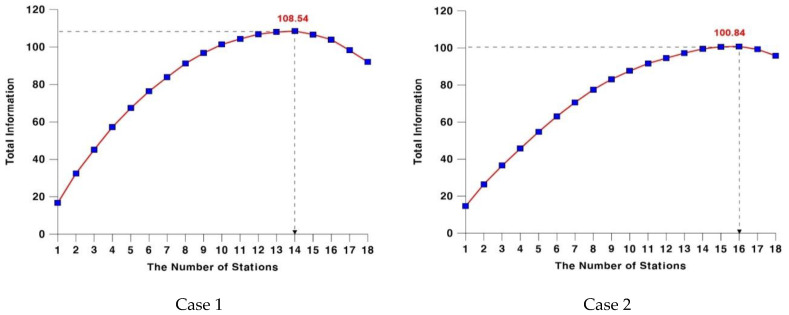
Comparison of the maximum information by each case.

In the study area, there were 18 stream gauge stations, and the maximum number of possible combinations from such stations (1–18) is thus 131,071. Table 7 and Figure 5 show changes in the maximum quantity of delivered information with a combination of the stations, based on the obtained information delivery matrix of the basin.

First, in Case 2, where the optimal stream gauge network was constructed with the empirical formula, 16 out of a total of 18 stations were selected. In Case 1, where the construction reflected upstream and downstream runoff characteristics, the maximum quantity of delivered information was obtained with 14 stations selected. It was noteworthy that Case 1 produced more information than Case 2, despite the fewer number of selected stream gauge stations. To be exact, Case 2 produced 100.84 of the maximum quantity of delivered information with 16 stations selected, while Case 1 generated 108.4 with 14 stations selected, which means fewer stations but more delivered information. If all the gauge stations are approached individually, the lack of connection between upstream and downstream leads to a result of a greater number of stations to achieve the maximum quantity of delivered information.

Thus, the upstream runoff characteristics have a direct influence on those of the lower part of the stream. In particular, in the case where two rivers are joined, the upstream runoff characteristics are under even more significant influence. If one of the two cases has the topographical nature of a mild slope, while the other has that of a steep slope, the runoff characteristics of the downstream would be determined by the topographical nature of each. However, when a junction is assumed as a single basin, these runoff characteristics of the upstream are hardly reflected. This research also supports the following. As Case 1 reflected the connection between the upstream and downstream gauge stations, more information was delivered, despite the fewer number of stations selected. This means that the selected stations can representatively provide the information delivered between upstream and downstream flows.

**Figure 6:**
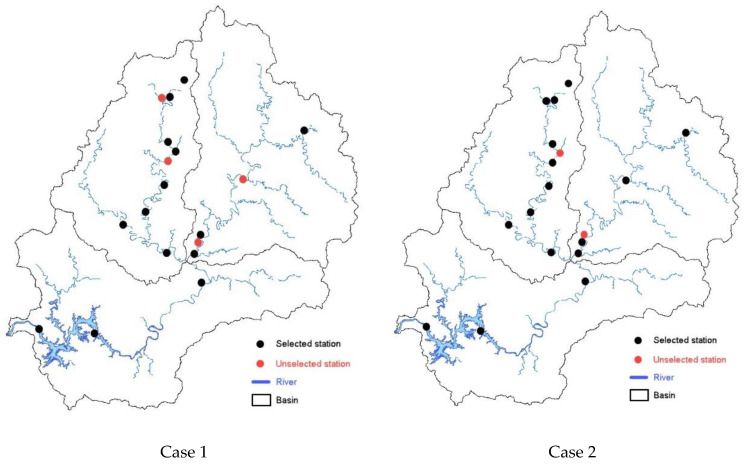
Comparison of optimal stream gauge stations for two methodologies.

Figure 6 shows the comparison result of the locations of stations selected for cases 1 and 2 with maximum information delivered. It is noted that 14 gauge stations are marked for Case 1, and 16 are marked for Case 2. The 12 stations (1, 5, 6, 7, 9, 12, 13, 14, 15, 16, 17, and 18) were selected for both cases 1 and 2. Of the existing stations at the Namhan River basin, three were selected for Case 1, and two were selected for Case 2; at the Pyeongchang River basin, nine were selected for Case 1, and nine were selected for Case 2; while at the Chungju Dam basin, three were selected for both cases 1 and 2. It was found that Case 1 provided more information delivered with a fewer number of stations compared to those in Case 2. As explained earlier, this is because the case reflects the upstream and downstream runoff characteristics. The ratio of selected stations was smaller for the Pyeongchang River basin (8 of 10, 80%) than for the Namhan River basin (3 of 5, 60%). This may be because the Chungju Dam basin better represents the characteristics of the Namhan River basin than those of the Pyeongchang River basin. Conversely, the Pyeongchang River basin requires more stations, because it represents complex runoff characteristics due to its topographical influence. A greater number of stations are located in the Pyeongchang River basin compared to the Namhan River basin.

This study targeted a limited range of existing stream gauge stations instead of having additional new stations when constructing an optimal stream gauge network. Therefore, the purpose of assessing stations in this study is to propose an optimal number and locations of stations for the basin. In addition, the study focused on designing an optimal stream gauge network that reflects the hydrological characteristics that each of the stations have. In this regard, there was a limitation in the direct assessment of an individual station installed for specific purposes such as irrigation, flood control, or other environmental objectives. However, the study can provide basic but important data for network integration for the effective hydrological management of stream gauge stations.

## 6. Conclusions

The study assessed optimal stream gauge networks that reflect the runoff characteristics of stations that are either upstream or downstream instead of approaching stations individually. The theory of entropy was applied to the study area, Korea’s Chungju Dam basin, for the construction of an optimal stream gauge network. Comparative analysis was conducted for the two cases where an optimal network was constructed. One was based on rainfall–runoff analysis, and another applied existing empirical formula to the construction. The unit hydrographs that were developed through rainfall–runoff analysis reflected the upstream and downstream runoff characteristics, and thus are more useful for constructing a gauge network, compared to the other case, which used empirical formulas. The reason for the greater usefulness is that the runoff characteristics of the downstream flow are under the influence of the upstream flow, and a change in upstream runoff determines downstream characteristics. In addition, this phenomenon was found at the junction of two rivers.

As a result of constructing optimal stream gauge networks using the two methodologies, the case without a connection between upstream and downstream required a greater number of gauge stations to secure the maximum quantity of delivered information. On the other hand, the case that reflects such a connection could acquire a greater maximum quantity of delivered information with a fewer number of stations. In addition, it was identified that the target area of the study, the Chungju Dam basin, requires 12 to 14 stream gauge stations out of the current total of 18 stations.

This study only considered changes in the quantity of delivered information when constructing optimal stream gauge networks, with a goal of providing hydrological data. Therefore, this study’s result would not be compatible with other purposes such as for irrigation, flood control, and other environmental or economic aspects. The reason for the greater usefulness is that the runoff characteristics of the downstream are under the influence of the upstream runoff, and a change in upstream runoff determines downstream characteristics. It is not easy to verify this. It can only be confirmed that Case 1 has the maximum information (108.54) on 14 observations, while Case 2 needs 16 observatories to reach the maximum information (100.84). Case 1 produced more information than Case 2, despite the fewer number of selected stream gauge stations. All the conditions of Case 1 and Case 2 are the same, and only the derivation process of the unit hydrograph is different. It may be due to the random error of Case 2 as well as the upstream and downstream runoff characteristics, which is one limit of this paper that we will discuss further in future study. Future study will relate radar correction and the importance of stations using multi-objective genetic algorithm. Research of the stream gauge network will also contribute to better understanding the water flows on the Earth’s surface and the connectivity of the flows.

## Figures and Tables

**Table 1:** Topographical characteristics and density of stream gauge stations in the study area.

Basin	No. of Stream Gauge Station	Name of Stream Gauge Station	Basin Area (km^2^)	Stream Length (km)	Stream Slope	Density	
						(km^2^/# R.G.)	(#R.G./km^2^)
Namhan River Basin	1	Imgye	457.5	7.5	0.01042	457.5	0.0022
	2	Jeongseon 2	1682.1	93.1	0.00569	841.1	0.0012
	3	Geoun	2272.1	142.2	0.00297	757.4	0.0013
	4	Yeongwol 2	2287.9	147.8	0.00263	572.0	0.0017
	5	Yeongwol	2447.8	153.4	0.00254	489.6	0.0020
Pyeongchang River Basin	6	Imokjeong	54.3	7.5	0.01604	54.3	0.0184
	7	Jangpyeonggyo	104.5	24.3	0.01214	52.3	0.0191
	8	Baekokpo	143.6	21.8	0.00590	47.9	0.0209
	9	Sanganmi	394.8	49.3	0.00790	98.7	0.0101
	10	Habanjeong	81.4	16.7	0.01420	81.4	0.0123
	11	Bangrimgyo	524.2	54.3	0.00726	104.8	0.0095
	12	Pyeongchang	695.6	76.9	0.00513	115.9	0.0086
	13	Panun	830.1	101.2	0.00348	118.6	0.0084
	14	Jucheon	528.8	73.2	0.00436	528.8	0.0019
	15	Yeongwol 1	1399.6	136.5	0.00250	140.0	0.0714
After confluence of Namhan and Pyeongchang River Basin	-	-	3847.0	-	-	256.5	0.0039
Chungju Dam Basin	16	Yeongchun	4543.4	181.5	0.00162	285.0	0.0035
	17	Cheongpung	5388.1	240.7	0.00126	316.9	0.0032
	18	Chungju Dam	6648.0	268.2	0.00126	369.3	0.0027
Total	-	-	6648.0	-	-	369.3	0.0027

**Table 2:** Recommended Minimum Densities of Stations (Area per Station) [23].

Physiographic Unit	Streamflow
Coastal	2750
Mountains	1000
Interior plains	1875
Hilly/undulating	1875
Small islands	300
Polar/arid	20,000

**Table 3:** Development of rainfall events for rainfall–runoff analysis. AMC: Antecedent Moisture Condition.

# Event	Date (Y/M/D/H)	Total Rainfall (mm)	Rainfall Duration (hr)	Total Five-Day Antecedent Rainfall (mm)	AMC	Maximum Rainfall Intensity (mm/hr)	Average Rainfall Intensity (mm/hr)	Centroid of Hyetograph (hr)
1	2008/07/23/10	220.4	92	141.4	III	15.62	2.40	32.6
2	2010/09/09/14	182.2	98	142.3	III	17.08	1.86	52.3

**Table 4:** Parameter estimation of lognormal distribution depending on two cases.

Basin	Number of Stream Gauge Station	Name of Stream Gauge Station	Case 1		Case 2	
			Average	SD	Average	SD
Namhan River basin	1	Imgye	1.43	0.44	0.92	0.18
	2	Jeongseon 2	2.15	0.41	1.77	0.53
	3	Geoun	2.55	0.36	2.68	0.62
	4	Yeongwol 2	2.59	0.35	2.82	0.68
	5	Yeongwol	2.58	0.36	2.58	0.57
Pyeongchang River basin	6	Imokjeong	1.47	0.40	1.00	0.28
	7	Jangpyeonggyo	1.62	0.40	0.62	0.46
	8	Baekokpo	1.51	0.39	0.72	0.44
	9	Sanganmi	1.74	0.38	1.19	0.50
	10	Habanjeong	1.81	0.46	0.97	0.24
	11	Bangrimgyo	1.80	0.41	1.23	0.48
	12	Pyeongchang	2.03	0.40	1.96	0.61
	13	Panun	2.23	0.37	2.73	0.68
	14	Jucheon	2.00	0.53	2.10	0.63
	15	Yeongwol 1	2.35	0.44	3.32	0.71
Chungju Dam basin	16	Yeongchun	2.54	0.43	3.05	0.58
	17	Cheongpung	2.83	0.38	3.75	0.64
	18	Chungjudam	3.57	0.25	3.80	0.63

**Table 5:** Information matrix for each of the stream gauge stations (Case 1).

	**1**	**2**	**3**	**4**	**5**	**6**	**7**	**8**	**9**	**10**	**11**	**12**	**13**	**14**	**15**	**16**	**17**	**18**
**1**	**5.203**	0.106	0.001	0.000	0.000	2.171	1.059	1.672	0.605	0.600	0.528	0.185	0.047	0.372	0.039	0.005	0.003	0.005
**2**	0.106	**5.133**	0.341	0.264	0.293	0.120	0.252	0.146	0.426	0.566	0.555	1.558	1.628	1.008	1.124	0.480	0.072	0.036
**3**	0.001	0.341	**5.002**	2.339	2.754	0.001	0.008	0.001	0.024	0.056	0.045	0.185	0.502	0.169	0.808	1.900	0.632	0.427
**4**	0.000	0.264	2.339	**4.974**	3.318	0.000	0.004	0.000	0.013	0.038	0.028	0.137	0.392	0.130	0.651	1.514	0.770	0.525
**5**	0.000	0.293	2.754	3.318	**5.002**	0.000	0.005	0.001	0.017	0.045	0.035	0.155	0.432	0.145	0.709	1.681	0.732	0.499
**6**	2.171	0.120	0.001	0.000	0.000	**5.108**	1.332	2.516	0.731	0.692	0.623	0.213	0.053	0.414	0.044	0.006	0.003	0.005
**7**	1.059	0.252	0.008	0.004	0.005	1.332	**5.108**	1.652	1.446	1.248	1.179	0.420	0.132	0.695	0.106	0.025	0.001	0.003
**8**	1.672	0.146	0.001	0.000	0.001	2.516	1.652	**5.083**	0.874	0.803	0.736	0.255	0.067	0.471	0.054	0.009	0.003	0.005
**9**	0.605	0.426	0.024	0.013	0.017	0.731	1.446	0.874	**5.057**	1.921	2.275	0.701	0.241	1.000	0.188	0.056	0.000	0.001
**10**	0.600	0.566	0.056	0.038	0.045	0.692	1.248	0.803	1.921	**5.248**	2.488	0.883	0.344	1.402	0.277	0.102	0.003	0.000
**11**	0.528	0.555	0.045	0.028	0.035	0.623	1.179	0.736	2.275	2.488	**5.133**	0.900	0.329	1.277	0.258	0.088	0.001	0.000
**12**	0.185	1.558	0.185	0.137	0.155	0.213	0.420	0.255	0.701	0.883	0.900	**5.108**	0.926	1.411	0.680	0.281	0.027	0.010
**13**	0.047	1.628	0.502	0.392	0.432	0.053	0.132	0.067	0.241	0.344	0.329	0.926	**5.030**	0.650	1.584	0.670	0.112	0.059
**14**	0.372	1.008	0.169	0.130	0.145	0.414	0.695	0.471	1.000	1.402	1.277	1.411	0.650	**5.389**	0.554	0.252	0.034	0.015
**15**	0.039	1.124	0.808	0.651	0.709	0.044	0.106	0.054	0.188	0.277	0.258	0.680	1.584	0.554	**5.203**	1.095	0.237	0.150
**16**	0.005	0.480	1.900	1.514	1.681	0.006	0.025	0.009	0.056	0.102	0.088	0.281	0.670	0.252	1.095	**5.180**	0.577	0.396
**17**	0.003	0.072	0.632	0.770	0.732	0.003	0.001	0.003	0.000	0.003	0.001	0.027	0.112	0.034	0.237	0.577	**5.057**	1.890
**18**	0.005	0.036	0.427	0.525	0.499	0.005	0.003	0.005	0.001	0.000	0.000	0.010	0.059	0.015	0.150	0.396	1.890	**5.002**

**Table 6:** Information matrix for each of the stream gauge stations (Case 2)**.**

	**1**	**2**	**3**	**4**	**5**	**6**	**7**	**8**	**9**	**10**	**11**	**12**	**13**	**14**	**15**	**16**	**17**	**18**
**1**	**4.331**	0.060	0.001	0.001	0.001	1.176	0.258	0.373	0.467	1.575	0.433	0.041	0.000	0.022	0.010	0.005	0.024	0.026
**2**	0.060	**5.389**	0.145	0.112	0.170	0.129	0.035	0.055	0.379	0.100	0.432	1.577	0.163	1.042	0.001	0.006	0.093	0.116
**3**	0.001	0.145	**5.546**	2.053	2.266	0.000	0.002	0.001	0.005	0.000	0.006	0.283	2.757	0.430	0.423	0.648	0.004	0.000
**4**	0.001	0.112	2.053	**5.638**	1.472	0.000	0.003	0.002	0.002	0.001	0.003	0.225	2.223	0.343	0.587	0.870	0.021	0.004
**5**	0.001	0.170	2.266	1.472	**5.462**	0.000	0.002	0.001	0.006	0.000	0.007	0.327	1.917	0.495	0.321	0.504	0.000	0.006
**6**	1.176	0.129	0.000	0.000	0.000	**4.742**	0.328	0.477	0.884	2.198	0.828	0.087	0.000	0.051	0.014	0.008	0.040	0.043
**7**	0.258	0.035	0.002	0.003	0.002	0.328	**5.248**	1.942	0.380	0.296	0.305	0.025	0.001	0.013	0.018	0.009	0.043	0.047
**8**	0.373	0.055	0.001	0.002	0.001	0.477	1.942	**5.203**	0.515	0.434	0.420	0.038	0.001	0.021	0.018	0.009	0.046	0.049
**9**	0.467	0.379	0.005	0.002	0.006	0.884	0.380	0.515	**5.331**	0.698	2.566	0.266	0.008	0.177	0.015	0.007	0.078	0.086
**10**	1.575	0.100	0.000	0.001	0.000	2.198	0.296	0.434	0.698	**4.589**	0.655	0.067	0.000	0.039	0.012	0.007	0.033	0.036
**11**	0.433	0.432	0.006	0.003	0.007	0.828	0.305	0.420	2.566	0.655	**5.290**	0.299	0.010	0.199	0.014	0.006	0.078	0.086
**12**	0.041	1.577	0.283	0.225	0.327	0.087	0.025	0.038	0.266	0.067	0.299	**5.530**	0.306	1.871	0.014	0.034	0.078	0.107
**13**	0.000	0.163	2.757	2.223	1.917	0.000	0.001	0.001	0.008	0.000	0.010	0.306	**5.638**	0.456	0.449	0.665	0.006	0.000
**14**	0.022	1.042	0.430	0.343	0.495	0.051	0.013	0.021	0.177	0.039	0.199	1.871	0.456	**5.562**	0.038	0.074	0.058	0.088
**15**	0.010	0.001	0.423	0.587	0.321	0.014	0.018	0.018	0.015	0.012	0.014	0.014	0.449	0.038	**5.682**	1.446	0.328	0.229
**16**	0.005	0.006	0.648	0.870	0.504	0.008	0.009	0.009	0.007	0.007	0.006	0.034	0.665	0.074	1.446	**5.479**	0.154	0.094
**17**	0.024	0.093	0.004	0.021	0.000	0.040	0.043	0.046	0.078	0.033	0.078	0.078	0.006	0.058	0.328	0.154	**5.578**	2.137
**18**	0.026	0.116	0.000	0.004	0.006	0.043	0.047	0.049	0.086	0.036	0.086	0.107	0.000	0.088	0.229	0.094	2.137	**5.562**

**Table 7:** Results of the optimized stream gauge network (Case 1 and Case 2).

Case 1			Case 2		
No. of Stations	Optimized Combination of Stream Gauge Stations	Maximum Information Content	No. of Stations	Optimized Combination of Stream Gauge Stations	Maximum Information Content
1	10	16.72	1	13	14.60
2	5,10	32.45	2	9,13	26.46
3	5,8,10	45.19	3	3,9,12	36.51
4	2,5,8,10	57.29	4	3,9,10,12	45.72
5	5,6,11,14,15	67.36	5	3,4,9,10,12	54.72
6	2,5,6,9,10,16	76.30	6	3,4,9,10,12,17	63.09
7	2,5,6,7,11,14,16	83.89	7	3,4,8,9,10,12,17	70.62
8	2,5,6,7,11,14,16,17	91.20	8	3,4,8,9,10,12,15,18	77.51
9	2,3,4,6,7,11,14,15,18	96.78	9	2,3,4,8,9,10,14,15,18	83.15
10	1,2,3,4,8,9,10,14,15,18	101.40	10	1,2,3,4,6,7,9,14,15,18	87.60
11	1,2,5,8,9,10,14,15,16,17,18	104.31	11	1,2,5,6,7,9,13,14,15,16,18	91.65
12	1,3,5,7,8,11,12,13,14,15,17,18	106.86	12	1,2,5,6,7,9,13,14,15,16,17,18	94.52
13	1,3,5,6,7,9,10,12,13,14,15,17,18	107.99	13	1,2,5,6,7,8,11,13,14,15,16,17,18	97.25
14	1,3,5,6,7,9,10,12,13,14,15,16,17,18	108.54	14	1,2,4,5,6,7,8,11,13,14,15,16,17,18	99.53
15	1,2,3,4,6,7,9,10,12,13,14,15,16,17,18	106.69	15	1,2,4,5,6,7,8,11,12,13,14,15,16,17,18	100.64
16	1,2,3,4,6,7,8,9,10,12,13,14,15,16,17,18	103.98	16	1,2,4,5,6,7,8,9,11,12,13,14,15,16,17,18	100.84
17	1,2,3,4,5,6,7,8,9,11,12,13,14,15,16,17,18	98.24	17	1,2,4,5,6,7,8,9,10,11,12,13,14,15,16,17,18	99.28
18	1,2,3,4,5,6,7,8,9,10,11,12,13,14,15,16,17,18	92.02	18	1,2,3,4,5,6,7,8,9,10,11,12,13,14,15,16,17,18	95.80
